# The Effectiveness of U.S. Public Health Surveillance Systems for Situational Awareness during the 2009 H1N1 Pandemic: A Retrospective Analysis

**DOI:** 10.1371/journal.pone.0040984

**Published:** 2012-08-22

**Authors:** Michael A. Stoto

**Affiliations:** 1 Department of Health Systems Administration, School of Nursing and Health Systems Administration, Georgetown University, Washington, District of Columbia, United States; Centers for Disease Control and Prevention, United States of America

## Abstract

**Background:**

The 2009 H1N1 outbreak provides an opportunity to learn about the strengths and weaknesses of current U.S. public health surveillance systems and to identify implications for measuring public health emergency preparedness.

**Methodology/Principal Findings:**

We adopted a “triangulation” approach in which multiple contemporary data sources, each with different expected biases, are compared to identify time patterns that are likely to reflect biases versus those that are more likely to be indicative of actual infection rates. This approach is grounded in the understanding that surveillance data are the result of a series of decisions made by patients, health care providers, and public health professionals about seeking and providing health care and about reporting cases to health authorities. Although limited by the lack of a gold standard, this analysis suggests that children and young adults are over-represented in many pH1N1 surveillance systems, especially in the spring wave. In addition, the nearly two-month delay between the Northeast and the South in the Fall peak in some surveillance data seems to at least partially reflect regional differences in concerns about pH1N1rather than real differences in pH1N1 infection rates.

**Conclusions/Significance:**

Although the extent of the biases suggested by this analysis cannot be known precisely, the analysis identifies underlying problems with surveillance systems – in particular their dependence on patient and provider behavior, which is influenced by a changing information environment – that could limit situational awareness in future public health emergencies. To improve situational awareness in future health emergencies, population-based surveillance systems such as telephone surveys of representative population samples and seroprevalence surveys in well-defined population cohorts are needed.

## Background and Objectives

In the Spring of 2009, a novel H1N1 influenza virus, now denoted pH1N1, emerged in North America and spread to the rest of the world in less than two months [1]. In the United States, where some of the first cases emerged, one of the public health challenges was to track the spread of the new virus and characterize its epidemiologic properties in order to guide and monitor the effects of response efforts. Existing public health surveillance and laboratory systems rapidly developed a case definition and new testing procedures, but public health laboratories soon became overloaded with samples to be tested, and surveillance definitions and procedures were changed as necessary to handle the load. Later in the year, as the epidemiological characteristics of the new H1N1 subtype emerged, traditional influenza surveillance systems were augmented with new systems tailored to the emerging epidemiology [2]. Some of these new systems could be described as syndromic surveillance approaches, which by using pre-diagnostic data, were thought to have a distinct advantage over the traditional surveillance method in terms of timeliness [3].

The epidemiology of pH1N1 has been well described elsewhere [4], [5], and adding to this understanding is not the goal of this paper. Rather, our purpose is to learn from the 2009 H1N1 experience about the strengths and weaknesses of current U.S. public health surveillance systems and to identify implications for measuring public health emergency preparedness. To do this we focus on two critical issues. First, we address the widely-held perception that children and young adults were at “higher risk.” Second, we assess the validity and utility of syndromic surveillance systems that were promoted by the President's Council of Advisors on Science and Technology (PCAST) [3] and other authorities. Both of these questions relate to the ability of public health surveillance systems to provide “situational awareness,” critical information needed to respond to disease outbreaks and other public health emergencies. This includes numbers of cases and other traditional surveillance data as well as information on critical response resources, medical care capacity, environmental threats, and public awareness [6].

We begin with a discussion of the data and methods employed in this paper, including indicators of the information environment during 2009 and how that might have biased the available surveillance data. We then review (1) the evidence regarding differential age-specific risks associated with pH1N1 and (2) surveillance systems' ability to accurately monitor pH1N1 cases over time. The discussion section addresses the limitations of this study, the implications of this analysis regarding both the strengths and weaknesses of current surveillance systems and alternatives that should be considered for the future, and the implications of this analysis for the measurement of public health preparedness.

## Results

### The information environment

**Figure 1:**
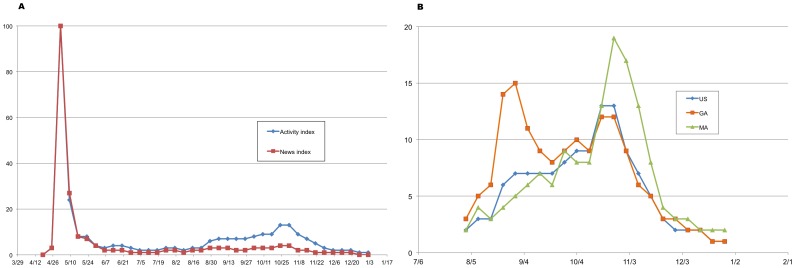
Google “Swine Flu” indices for the Northeast, South and entire United States Panel A. Google Insights “Swine Flu” activity and news indices, U.S., April 12, 2009–January 2, 2010. Panel B. Google Insights “Swine Flu” activity index, U.S., Georgia (GA), and Massachusetts (MA), August 2, 2009–January 2, 2010. Source: Google Insights search for “swine flu” at http://www.google.com/insights/search/#q=SWINE%20FLU&geo=US&date=1%2F2009%2012m&cmpt=q.

By way of background for the subsequent analyses, Figure 1 Panel A represents trends in the number of U.S. Google searches for “swine flu” between April and December of 2009 on a scale of 0 to 100. Both the number of general searches (labeled as “Activity index”) and news searches (“News index”) both grow rapidly from 0 in mid-April to 100 in the week ending May 2, 2009, corresponding to a rapid set of announcements from the Centers for Disease Control and Prevention (CDC) and the WHO about the emergence of the pandemic. Both indices drop off by the end of May. The general searches, but not the news searches, pick up again at the end of August, reaching a peak of 13 on a scale of 0 to 100 at the end of October, and drop off to zero by the end of the year.

Figure 1 Panel B presents the Google Insights Activity index for the United States as well as for Massachusetts and Georgia, populous and presumably typical states in CDC Regions 1 (Northeast) and 4 (the South) respectively. In the south, “swine flu” searches peaked at the end of August at 15 and again at 12 in mid-October. In the Northeast, on the other hand, the activity index peaked at 19 at the end of October, higher and a week later than the United States as a whole.

### Age-related risks of pH1N1

One of the most commonly held perceptions about pH1N1 is that children and young adults are at especially “high risk.” For instance, on May 17, 2009 in an article entitled “Age of Flu Victims Has Big Implications,” the *Washington Post* reported that “perhaps the most worrisome features so far are the number and severity of cases in teenagers and young adults. This was noticed early, and the pattern has not changed much now that there are 5,000 laboratory-confirmed infections and probably more than 100,000 overall. The average age of the confirmed and probable cases is 15 years. Two-thirds are younger than 18.” [7] Similarly, an August 2009 PCAST report said that confirmed cases were concentrated in younger age groups, up to age 24, almost all severe cases were in people younger than age 65, and the consequences of infection in this epidemic were already known to be far more severe for children and young adults, and seemingly milder for people over age 65 [3].

With seasonal influenza, serious illness requiring hospitalization and death are uncommon in children (except for infants) and young adults, so this pattern has important public health implications. Indeed the fact that the first cases of pH1N1 that came to light in southern California, Mexico, and in New York City were in children and young adults was an important clue that a new pandemic viral subtype had emerged [8]. The perception, based on these reports, that children and young adults were “at risk” also led to school closings in the Spring of 2009 [9], [10], the issuance of recommendations for schools, universities, and day care centers [11], and recommendations that children and adolescents be given priority for immunizations [12]. The same assumption also influenced recommendations from the WHO [13].

Regarding the other end of the age spectrum, on May 22, 2009 CDC reported the early results of an antibody study indicating that children had no existing cross-reactive antibody to pH1N1, while about one-third of adults older than 60 years of age had such a reaction. These results were attributed to the possibility that older people had been previously exposed, either through infection or vaccination, to an influenza A(H1N1) virus that was more closely related to pH1N1 than contemporary seasonal influenza viruses [14].

Typical of the information available early in the pandemic, a report from CDC published ahead of print by the *New England Journal of Medicine* on May 7, 2009 noted that 60% of 642 confirmed cases were 18 years of age or younger [15]. Similarly, in a report on 272 patients who were hospitalized with laboratory-confirmed pH1N1 from April to mid-June 2009 found that 45% were under the age of 18 and 5% were 65 years of age or older [16]. Another early study, published in CDC's Morbidity and Mortality Weekly Report in late August, reported that the attack rate was highest among children aged 5–14 years (147 per 100,000 population), 14 times higher than the rate for adults aged 60 and older [17]. Shortly afterwards, a CDC pediatric deaths surveillance study reported that children under 18 years of age represented 36 of the 477 laboratory-confirmed pH1N1 deaths through early August [18].

**Figure 2:**
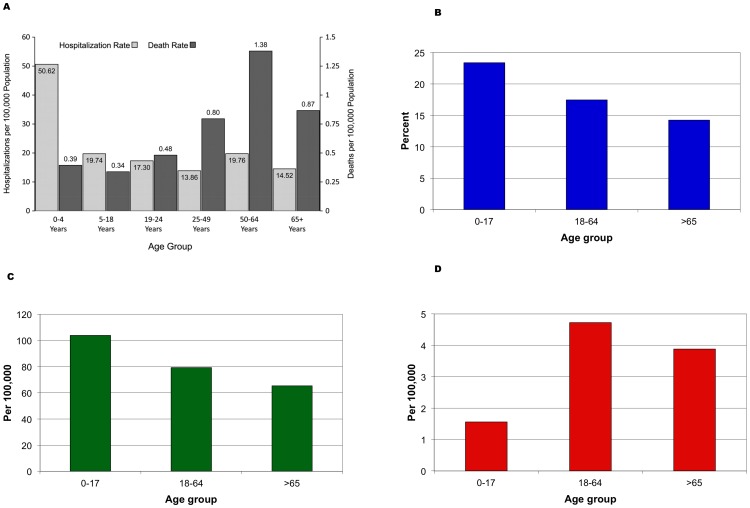
pH1N1 infection, hospitalization, and death rates. Panel A. Aggregate hospitalization and death reporting activity (AHDRA) hospitalization and death rates per 100,000 population by age group, laboratory-confirmed pH1N1 influenza infection—United States, August 2009–February 2010. Source: Jhung, 2011. Panel B, C & D. 2009 H1N1-Related Deaths, Hospitalizations and Cases, U.S. April 2009–January 16, 2010. Author's calculations based on CDC EIP program estimates: Updated CDC estimates of 2009 H1N1 influenza cases, hospitalizations and deaths in the United States, April 2009–April 10, 2010 Available from: http://www.cdc.gov/h1n1flu/estimates_2009_h1n1.htm.

National, population-based hospitalization and mortality rates did not become available until after CDC established AHDRA in August 2009. Using data from the states reporting laboratory-confirmed cases, CDC reported that the highest rates of hospitalizations were observed among the 0–4-year-old age group, which had rates 2- to 3-fold higher than those observed in the other age groups (see Figure 2 Panel A). In addition, the majority of hospitalizations (>70%) reported were in patients less than 50 years of age and fewer than 10% were in patients 65 years of age or older [4]. Using data from AHDRA, CDC reported that “the age distribution of laboratory-confirmed pH1N1 influenza–associated death rate was markedly different from that seen in typical influenza seasons. In contrast to typical influenza seasons, when 90% of deaths occur in the elderly population, 86% of pH1N1 deaths reported to AHDRA were in persons under 65 years of age, with the highest rates found in persons aged 50–64 years” (Figure 2 Panel C) [4].

To properly understand the risks, one must first recognize that there are two different risks in question: incidence (the risk that someone becomes infected) and severity (the risk of suffering consequences such as severe illness requiring hospitalization or even death). Based on data from the CDC EIP program [19], Figure 2 Panel B presents the attack rate as well as the population-based hospitalization and death rates by age. The age-specific attack rates, which reflect the percentage of the population infected at some point during the pandemic, do bear out the elevated risk to children (23.4% for children under age 18 and 17.5% for adults aged 18–64 vs. 14.2 percent for those aged 65 and older). The hospitalization rates show a similar pattern: 104.0 per 100,000 for children under age 18, 79.2 per 100,000 for adults aged 18–64, and 65.4 per 100,000 for those aged 65 and older. While the attack and hospitalization rates are higher for children than for adults, the rates for children are less than twice that for seniors, not quite as dramatic as some of the figures presented at the beginning of this section. Death rates, on the other hand, are substantially higher for adults aged 18–64 than for those under 18 (4.72 vs. 1.56 per 100,000), with the rate for 65 and older population at an intermediate level of 3.88 per 100,000.

These rates, presented in Figure 2 Panels B, C, and D, however, are all based on certain assumptions that must be examined. The basis for the estimates is the number of influenza-associated hospitalizations reported in CDC's EIP program, which covers 62 counties in 10 states, not the number of pH1N1 cases, hospitalizations, and deaths reported to state and local health officials (individual case reporting was discontinued in May 2009 when laboratory capacity proved insufficient to handle the surge in number of potential cases). Reed and colleagues estimate these multipliers based on a variety of data relating to both seasonal and pH1N1 influenza, but assume that they are the same for every age group and constant over time (except that they account for the change in testing recommendations on May 12) [20]. The number of incident cases is estimated as a constant multiple of the number of confirmed cases, which explains why the estimated age-specific attack and hospitalization rates have similar patterns. The multipliers are based on estimates of the fraction of influenza cases in which health care is sought, specimens are tested, and the tests are positive [20].

However, by mid-May, the number of clinical samples being submitted to state labs for testing became overwhelming, and CDC and state health departments recommended viral testing only if it would affect their care, and states no longer reported individual confirmed and probable cases to CDC [21]. Moreover, as case counts grew, aggregate reporting replaced individual case reports in most jurisdictions, and most symptomatic cases were not tested, confirmed, or reported, and the proportion tested varied geographically and over time [22]. Combined with the perceptions that children were at higher risk and older adults less so, it is possible that children and young adults with influenza-like illness (ILI) would be more likely to seek care and to be tested, and older adults less so. Population-based BRFSS data suggest that between September, 2009 and March, 2010, health care was sought by 56% of children with self-reported ILI, compared to 40% of adults [23], and it is possible that the differential was stronger earlier in the outbreak, when fears about the risks for children were more common.

Regarding the attack rate, BRFSS survey data suggest that between September, 2009 and March, 2010, the average monthly proportion of children under age 18 who reported having experienced ILI in the preceding 30 days was 28.4%, compared to 8.1% in adults [23]. These percentages vary by month, but at their peak in November (referring to illness in October and November) they were 35.9% for children and 20.4% for adults. No national data are available for the spring wave, but a survey conducted in New York City in mid-June found self-reported ILI prevalence rates of 20% and 22% in children aged 0 to 4 and 5 to 17 respectively, vs. 10% and 6% in adults aged 18 to 64 and 65 and older respectively [24].

With respect to hospitalizations and deaths, the patterns in Figure 2 Panel C based on EIP data are similar to those based on the AHDRA surveillance system summarized in Panel A. In reporting the AHDRA rates, Jhung and colleagues make the point that the age distributions are markedly different than those for seasonal influenza [4]. These CDC estimates do not, however, show that children have a higher risk of dying than adults. Indeed, if reckoned in terms of the case fatality rate, the proportion of cases who die of the disease, the risks would be skewed even more towards older groups since the denominator (the number infected) is lower. Moreover, following the announcement of the CDC antibody study suggesting that older adults has some residual protection against H1N1 viruses, it is possible that seniors with ILI might not have been tested and their subsequent pH1N1 related death not classified as such. Correcting for such an effect, about which there is no statistical evidence, would increase the pH1N1-associated death rate in the 65 and older age group.

Given concerns about the perceived severity of pH1N1 for children, parents might have been more likely to seek medical care for their children than in previous years or compared to adults with the same symptoms. McDonnell and colleagues find evidence of this effect, at least for the spring wave, in an analysis of data from an integrated hospital system operating 18 hospital EDs. During the week beginning April 27, 2009, the number of pediatric (18 years or younger) ED visits increased 19.7% from the previous week, compared to 1.0 percent for adult patients. In addition, the proportion of pediatric ED patients admitted to the hospital decreased 5.6 percentage points from 23.2% to 17.6%, while the adult admission rates were decreased only 0.6 percentage points from 13.3% to 12.7% [25]. In another study done by Dugas and colleagues, the Google Flu Trends web search queries were correlated with weekly visits at the pediatric ED but not the adult ED during the three waves of pH1N1 peaks [54]. Since pH1N1 was very much in the news in this week but there were not likely to be many actual cases in the hospital system's population, these findings suggest that the increase in pediatric ED cases represents parent over-reaction.

**Figure 3:**
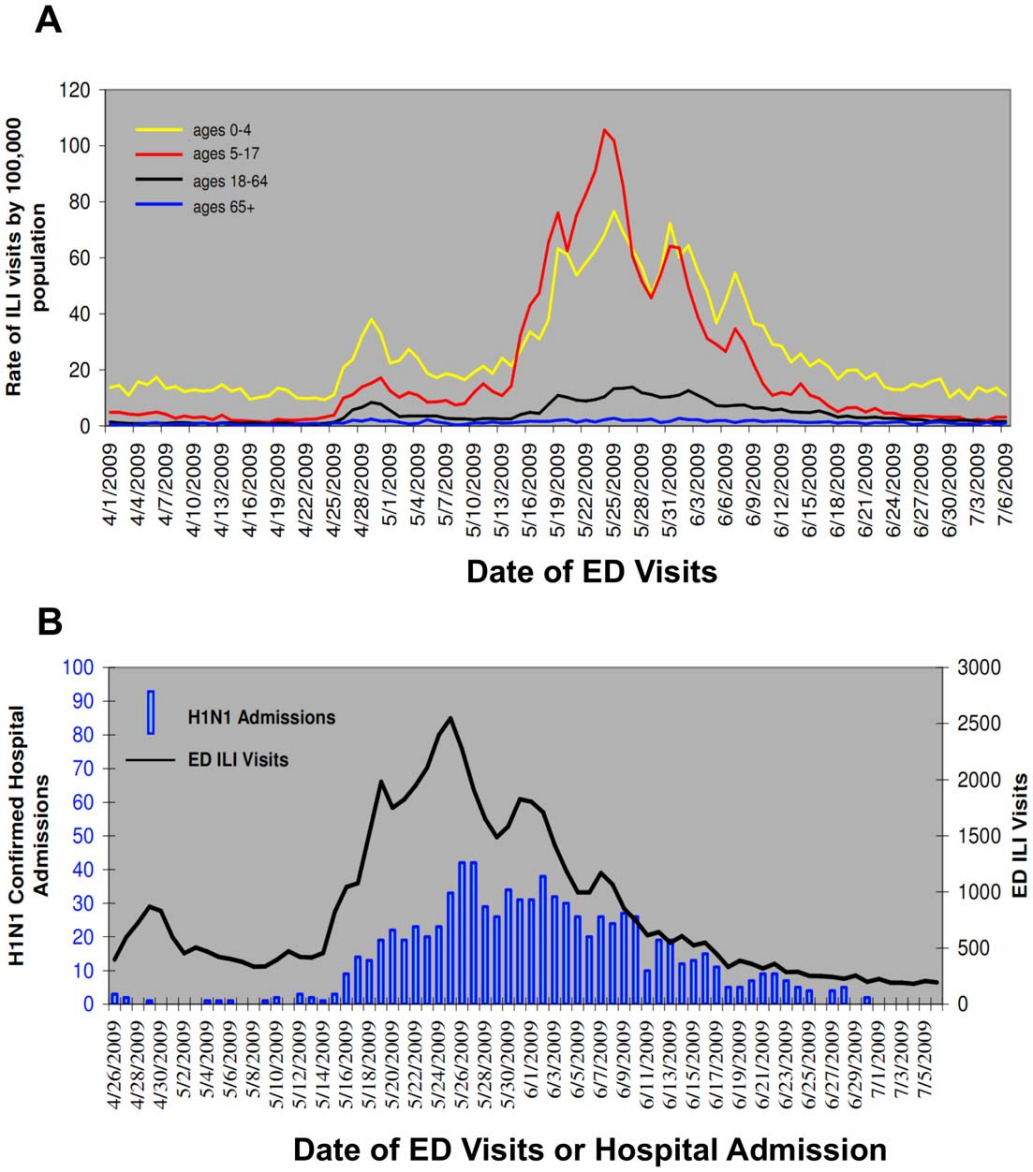
Influenza-related emergency department visits and hospitalizations. Panel A. Rate of ILI syndrome visits (based on chief complaint) to New York City emergency departments by age group, April 1, 2009–July 6, 2009. Panel B. Laboratory-confirmed H1N1 hospital admissions and emergency department (ED) Visits for ILI in New York City, April 1, 2009–July 6, 2009. Source: New York City Department of Health and Mental Hygiene Health Alert #27: Pandemic (H1N1) 2009 influenza update, revised reporting requirements and testing procedures Available from: http://www.nyc.gov/html/doh/downloads/pdf/cd/2009/09md27.pdf.

A similar effect can be seen in ED utilization and hospital admission rates in New York City [26]. As shown in Figure 3 Panel A, pediatric ED visits with ILI as a chief complaint peaked in late April, following the news about pH1N1 cases in high school students who had travelled to Mexico and their class mates, and rose sharply around May 15, when the global pandemic was prominent in the news. Figure 3 Panel B, however, indicates that laboratory-confirmed hospital admissions did not peak until May 26, suggesting that some ED visits earlier in the month reflected parental concern rather than pH1N1 infection.

A comparison of data from EIP coverage areas in New York State with an alternative sentinel hospital program (SHP) surveillance system established to monitor pH1N1, however, shows the opposite effect. Analyzing data from October 1, 2009 through March 31, 2010, Noyes and colleagues note that the age distribution of confirmed cases is very different in the two surveillance systems: children comprise 59% of SHP admissions but only 27% of EIP admissions [27]. These figures suggest that children might be at a *higher* risk of pH1N1-related hospitalization that the CDC EIP estimates suggest. However, the two surveillance systems differ in a number of ways that might effect this comparison: they cover different counties within the state, and the SHP program includes only data from a single surveillance hospital in a given area compared to the comprehensive coverage of the EIP. Perhaps more importantly, according to the authors, is the dependence on provider-driven testing practices. The EIP's nationally-defined protocol might have missed cases presenting with atypical clinical presentations or whose rapid influenza test (which is known to have a low sensitivity for pH1N1) was a false negative. The SHP, on the other hand, tested all patients admitted with ILI [27]. It is also possible that these factors differed between children and adults. Another dimension is the time period covered. The CDC EIP estimates refer to April 2009 through January 16, 2010, with most cases distributed in roughly equal parts between the spring wave (April through July) and the fall wave (August through November). The New York analysis, on the other hand, covers October 1, 2009 through March 31, 2010, and finds that the SHP data include a sustained number of ILI admissions in December and January.

Thus, the picture about the age-specific risks of pH1N1 that emerges from these analyses is mixed. With respect to the risk of infection per se, it does appear that children were more likely than adults to report ILI in population-based surveys, but these suggest that the ratio is closer to 2 to 1 than 14 to 1 (to cite an extreme example [17]). The age-specific rate of hospitalization for pH1N1 influenza also seems to be higher for children than adults, falling from 104.0 per 100,000 for ages 0–17 years to 64.4 per 100,000 for ages 65 and older (see Figure 2). However, these estimates are highly dependent on unverified assumptions and there are indications that these rates are and may have varied substantially during the pandemic, so the apparently higher rates in children, less than a 2 to 1 ratio, are somewhat uncertain. Finally, with respect to mortality rates, CDC's estimate for the 18 to 64 age group is more than 3 times as high as for children under age 18 (4.72 vs. 1.56 per 100,000). The population-based mortality rate for age 65 and older is less than for age 18 to 64, but more than twice that for children (3.88 vs. 1.56 per 100,000), and it is possible that the 65 and older rate is an underestimate due to under-reporting.

### Monitoring pH1N1 cases over time

It is generally accepted that pH1N1 influenza activity in the United States occurred in two distinct waves, the first peaking in June 2009 and the second in October 2009 [2]. However, the specific timing of these waves, and how the timing may have varied in different regions of the country, is less certain. In particular, trends and patterns in surveillance data may be related to awareness – both by patients and healthcare providers – about the risks of pH1N1.

**Figure 4:**
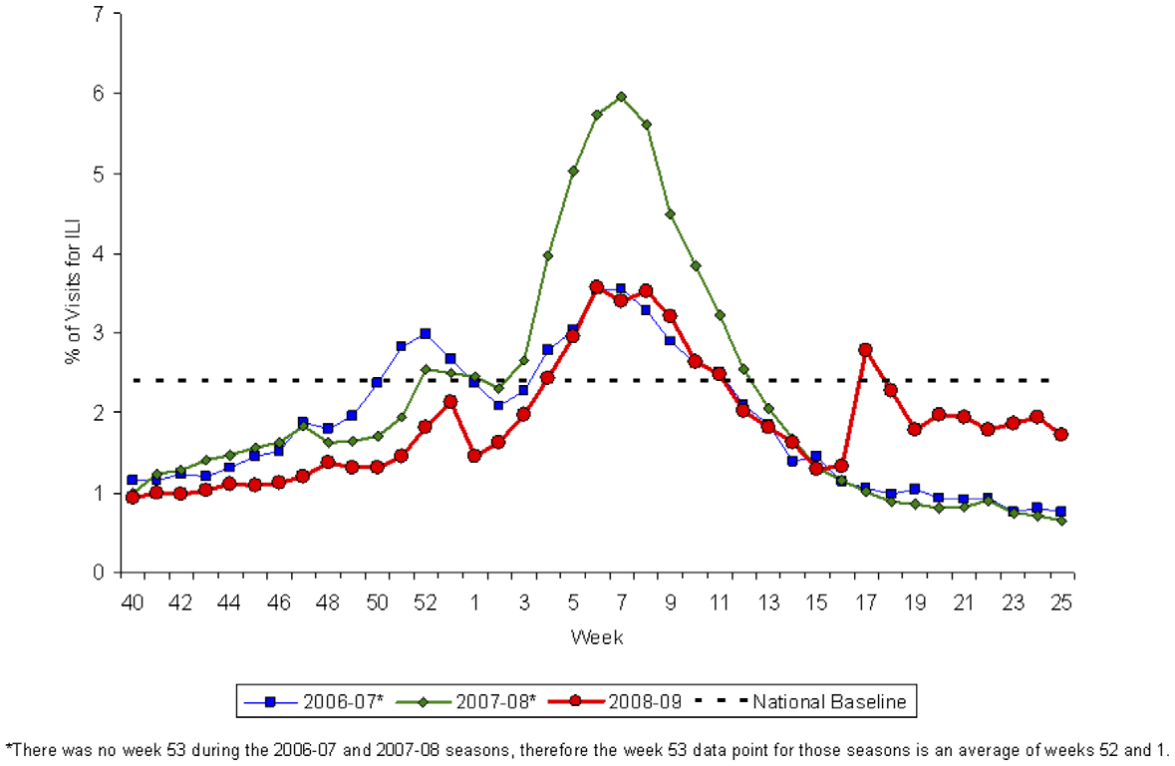
Outpatient influenza-like illness surveillance. Percentage of visits for ILI Reported by the U.S. Outpatient Influenza-like Illness Surveillance Network (ILINet), National Summary 2008–09 and Previous Two Seasons. Source: CDC Flu View, 2008–2009 Influenza Season Week 25 ending June 27, 2009. Available from: http://www.cdc.gov/flu/weekly/weeklyarchives2008-2009/weekly25.htm.

**Figure 5:**
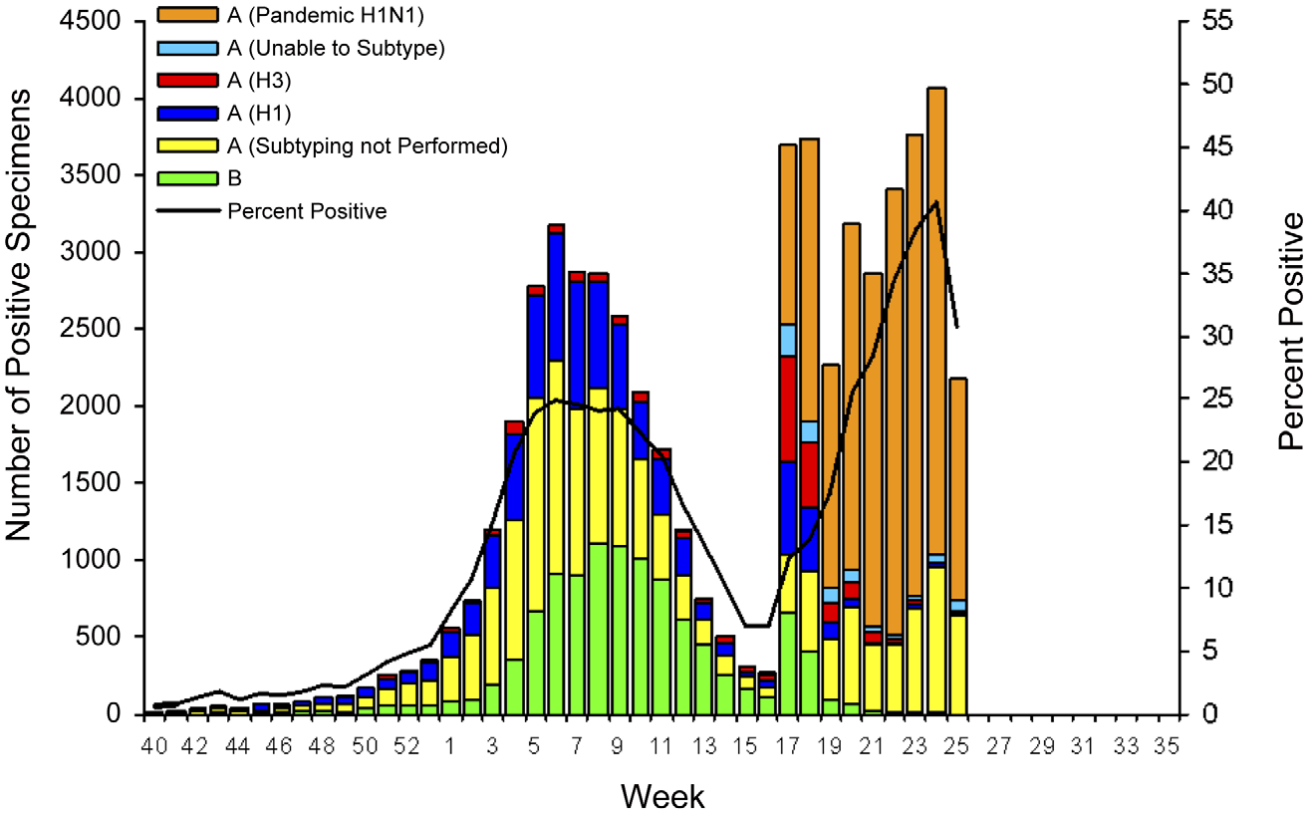
Laboratory-confirmed influenza cases reported to CDC by U.S. WHO/NREVSS collaborating laboratories, national summary, 2008–09. Source: CDC Flu View, 2008–2009 Influenza Season Week 25 ending June 27, 2009. Available from: http://www.cdc.gov/flu/weekly/weeklyarchives2008-2009/weekly25.htm.

To explore this hypothesis, we first consider surveillance reports from the spring pandemic wave with data from two of the key near real-time surveillance systems as they appeared at the end of June, 2009, about two months into the spring wave [28]. By convention, the weeks are numbered starting at the beginning of January. Figure 4 presents data from CDC's ILINet system, reflecting the proportion of outpatient visits for influenza-like illness. The proportion of visits for ILI increased sharply in Week 17, which ended on May 2, 2009, to 2.8%, a level that exceeds the national baseline and is more characteristic of the winter months. Figure 5, which displays the number and percentage of respiratory specimens in the United States testing positive for influenza reported by WHO/NREVSS collaborating laboratories, also shows a dramatic increase in the number of positive specimens – represented by the height of the bar – for Week 17. These peaks correspond temporally to a rapid series of announcements, starting on Tuesday April 21 of human-to-human transmission in California of a new viral sub-type, of a major outbreak in Mexico, of confirmed cases in New York City students who had travelled to Mexico, confirmation of world-wide spread of the new virus leading to an increase in the WHO's pandemic alert level, and closing of approximately 300 U.S. schools. Note that Figure 3 shows a similar peak at the end of April in New York City hospital ED visits for ILI.

One possible explanation for these peaks is that they reflect the rapid spread of ILI throughout the United States. It is also possible, however, that they reflect increased concern about the pandemic, leading people to seek health care for symptoms they might otherwise treat themselves, and physicians to send clinical samples in for testing. Indeed the Google Insights activity index, as shown in Figure 1, grew from 0 to 100 during this time, and never rose to more than 15 throughout the rest of 2009.

Moreover, a closer examination of Figure 5 shows that it is the number of samples submitted, not the number positive, that accounts for the sharp increase in Week 17. Of the 3911 samples tested in Week 16, 7.1% were positive for influenza. In Week 17, the number of samples submitted grew to 30,020, but only 12.3% were positive for influenza. And excluding the specimens that were determined to be influenza A but sub-typing was not done, at most 4.6% of the samples submitted in Week 17 were possibly pH1N1 (i.e. either determined to be novel H1N1 or unsubtypable influenza A). As shown by the black line in Figure 5, the proportion positive for influenza grows to about 40% in June, but the maximum proportion of samples submitted that might be pH1N1 never exceeds 35% (based on the author's calculations and not shown in the figure). Since the proportion of submitted samples that can possibly be pH1N1 was so low in Weeks 17 and 18, it seems likely that the sharp increase depicted in Figure 5 does not represent rapid spread of the pandemic virus in the U.S. population but rather increased awareness on the part of patients in seeking care and physicians in sending clinical samples for testing.

**Figure 6:**
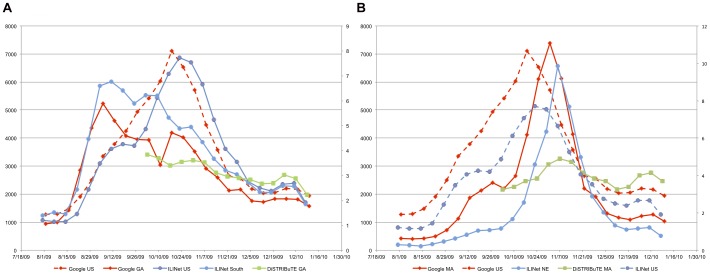
Influenza-like illness surveillance for the Northeast, South and entire United States. Panel A. Percentage of visits for influenza-like illness (ILINet), U.S. and South, July 31, 2009–January 8, 2010; Google Flu Trends index, U.S. and Georgia (GA), August 2, 2009–January 10, 2010; scaled DiSTRIBuTE ILI trends, Georgia (GA), October 3, 2009–January 9, 2010. Panel B. Percentage of visits for influenza-like illness (ILINet), U.S. and Northeast (NE), July 31, 2009–January 8, 2010; Google Flu Trends index, U.S. and Massachusetts (MA), August 2, 2009–January 10, 2010; scaled DiSTRIBuTE ILI trends, Massachusetts (MA), October 3, 2009–January 9, 2010. Sources: author's calculations based on CDC, Google, and DiSTRIBuTE data available from: http://www.cdc.gov/h1n1flu/cdcresponse.htm, http://www.google.org/flutrends/, and http://isdsdistribute.org/moreinfo.php.

The fall wave presents an opportunity to assess the ability of surveillance systems to monitor regional trends pH1N1. In particular, it was widely noted that the fall wave began earlier in the South and later in the Northeast. This pattern can be seen clearly in the ILINet data in Figure 6, and also in percent of samples submitted to WHO/NREVSS that were positive for influenza (not shown). The patterns in Google Flu Trends data, thought to be a proxy for the number of people with flu symptoms, for the same period (but using Georgia and Massachusetts as proxies for the regions, since the data are available for selected states and not region) are remarkably similar. However, DiSTRIBuTE project ED surveillance data from the same states (which are only available starting in October) display a somewhat different pattern. In both Georgia and Massachusetts, the difference between the maximum and minimum is much less than in either the ILINet or Google Flu Trends data. The Georgia DiSTRIBuTE data drop throughout the entire period, as do the ILINet or Google Flu Trends data. The Massachusetts DiSTRIBuTE data, on the other hand, peak shortly after either the ILINet or Google Flu Trends data, and again in late December.

There are two possible explanations for the patterns seen in Figure 6, both of which could be operating simultaneously. One hypothesis is that the fall wave actually did peak earlier in the South, and indeed the difference in timing has been related to differences in school starting dates [29]. The Northeast peak lags behind the South's by about two months, however, much longer than the difference in school start times. Moreover, it seems highly unlikely that a pandemic virus capable of spreading around the world in a matter of weeks would take two months to travel up the east coast of the United States from the South to the Northeast. The alternative hypothesis is that both the ILINet and Google Flu Trends data both reflect regional concerns about pH1N1, based in part on the local media. This hypothesis is supported by seeing the same regional patterns in the Google Insights “Swine Flu” activity index (Figure 1 Panel B), which is intended to reflect general searches rather than searches based on one's own symptoms. Furthermore, a two-week delay between the ILINet peak in the Northeast and the peaks in both Google data sources for Massachusetts further suggests that the ILINet peak represents people seeking care out of concern about pH1N1 rather than symptoms alone. Thus, although there could be some real differences in timing between the South and the Northeast, it seems likely that ILINet and Google Flu Trends data at least partially reflect regional differences in concerns about pH1N1.

## Discussion

From a systems perspective, public health surveillance data are the product of a series of decisions made by patients, health care providers, and public health professionals about seeking and providing health care and about reporting cases or otherwise taking action that comes to the attention of health authorities. And all of these decisions are potentially influenced by what these people know and think. Outpatient, hospital-based, and ED surveillance systems, for instance, all rely on individuals deciding to present themselves to obtain health care, and these decisions are based in part on their interpretations of their symptoms. Even the number of Google searches and self-reports of ILI in the BRFSS survey can be influenced by individuals' interpretation of the seriousness of their symptoms. Virologic surveillance and systems based on laboratory confirmations depend on physicians deciding to send specimens for testing. Every element of this decision-making is potentially influenced by the informational and policy environment (e.g. media coverage, current case definitions and practice recommendations, implementation of active surveillance), processing and reacting to the information on an individual level (e.g. health care seeker's self-assessment of risk, incentives for seeking medical attention and self-isolation; health care provider's ordering of laboratory tests), and technical barriers (e.g. communication infrastructure for data exchange, laboratory capacity). Because all of these factors change over time, so did the biases in the surveillance data, and as a result the U.S. public health system' ability to characterize and track the pH1N1 was compromised.

Although this analysis is limited by the lack of a gold standard – definitive knowledge of the actual number of pH1N1 cases and the characteristics of the individuals who are affected – a triangulation approach that compares multiple data systems, each with different expected biases over time, strongly suggests that children and young adults are over-represented in many pH1N1 surveillance systems, especially in the spring wave. In other words, children and young adults may not have been “at risk” to the extent that some thought, but rather the perception that they were led to surveillance results that supported this perception. And because ILINet, ED surveillance data, and other syndromic surveillance systems that depend on counts of people seeking care, especially for children, are influenced by the information environment, some apparent trends and geographic differences were, in part, reflections of what people thought was happening rather than actual numbers of cases. Comparing data from the spring and fall wave of pH1N1 in Wales, Keramarou and colleagues find a similar effect, with relatively more ILI visits in the Spring when media activity was intense and fewer in the Fall when media activity waned [30]. Chowell and colleagues also note that in Mexico the median age of laboratory-confirmed ILI cases was 18 years overall in 2009, but increased to 31 years during the fall wave. In France, ILI cases peaked in the first week of September (week 36), but according to virologic and clinical data the pandemic started in mid-October (week 44). Casalengo and colleagues [31] note that many of the early ILI cases were actually rhinovirus and other non-influenza viruses, and attribute the September increase in pediatric ILI cases to massive media coverage at the start of the school year and the general level of anxiety at that time.

Of course, epidemiologists recognize these potential biases and typically note them and present their analysis of the available data with appropriate caveats. But especially when the crisis is acute and need for information urgent, what seem like subtle methodological points to policymakers and the public can easily become lost. Even straightforward points can be confused. For instance, the dramatic increase in week 17 in the height of the colored bar in Figure 5 seems consistent with the alarmist headlines at that time and can easily be misinterpreted as a sudden increases in pandemic cases. In fact, as the analysis (and even just a careful reading of the graph) shows, this increase mainly reflects an increase in the number of samples submitted for testing rather than the percentage that are positive for pH1N1.

Confusion about whether children and young adults were more “at risk” for pH1N1 is compounded by different ideas about the meaning of risk. This term can mean the probability that one is infected, develop symptoms, requires hospitalization, or dies. Furthermore, the risk of severe consequences can be figured in terms of those who are infected or develop symptoms or in terms of everyone in some demographic group. Careful analysis of the data suggests that children and young adults do seem to have been more likely than older people to have been infected with pH1N1. On the other hand, the relative proportions of infected cases – or of the population – who die or require hospitalization may be higher for older adults. The pH1N1 surveillance literature uses nearly all of the possible definitions of risk, often with little clarify about which one is intended, and this further confuses public understanding of the situation. Minor differences among surveillance systems regarding standard age groupings, for instance, and differences in standard data presentation formats also adds to confusion.

The tendency to report cumulative case counts, especially in the early days of the pandemic, also complicated the interpretation of the data. By definition, cumulative case counts can only increase – they are not reduced as people regain their health or die after a course of illness – even if the incidence of new cases peaks. Cumulative counts also increase as new cases that occurred before epidemiologists were aware of the outbreak are discovered. Perhaps because case counts are a feature of other types of disasters, policy makers expect them in disease outbreaks, but presenting the data in this way can lead to misunderstandings.

This analysis benefits from hindsight, and is not intended as a criticism of the epidemiologists who had the much more difficult challenge of interpreting the data as the pandemic was emerging and spreading through the population. Indeed, the goal is not to question the epidemiology of pH1N1 but rather to learn from the U.S. experience about the performance of critical surveillance systems. In addition, this analysis adopts an outsiders' perspective, that is, we are not aware of all the information – or the pressures – that epidemiologists faced during the pandemic. While in some ways this is a limitation, in other respects it allows us to question some of the assumptions that, in the press of time and events, the public health officials dealing with pH1N1 may not have had the luxury of doing.

## Data and Methods

This analysis is a retrospective secondary analysis of a variety of data sources that were used to track pH1N1 in the United States in 2009, some of which were developed especially for this purpose. Many of these systems are operated by the CDC; these include systems for virologic surveillance and for tracking outpatient illness, influenza-associated hospitalizations, pneumonia- and influenza-related mortality, and influenza-associated pediatric deaths, and the geographic spread of influenza [2]. Other data are provided by state and local health departments as well as private sector organizations as described below.

The primary means for virologic surveillance is the WHO-affiliated National Respiratory and Enteric Virus Surveillance System (NREVSS), through which 140 public and private sector collaborating laboratories submit weekly information on the total number of respiratory specimens tested for influenza and the number positive by influenza type, subtype, and age group [2].

Outpatient illness surveillance is conducted by CDC's Influenza-Like Illness Surveillance Network (ILINet) system. ILINet receives weekly reports from more than 3,300 health care providers on the total number of patients seen for any reason and the number of patients with ILI, by age group, defined as a temperature of >37.8 C and cough and/or sore throat, in the absence of a known cause other than influenza [2]. In addition, since late 2009 CDC has partnered with the International Society for Disease Surveillance (ISDS) through the DiSTRIBuTE project (Distributed Surveillance Taskforce for Real-time Influenza Burden Tracking and Evaluation) to gather and analyze aggregate Emergency Department (ED) surveillance data from a number of state and local jurisdictions, each using its own definition of ILI [32]. Our analysis also utilizes ED data from an integrated health system operating 18 hospitals in a western state [25].

CDC, in collaboration with state health departments and academic centers in 10 states collects data on laboratory confirmed influenza-associated hospitalizations in children and adults through the Emerging Infections Program (EIP). This population-based surveillance is conducted in 60 counties that represent 7% of the U.S. population [2]. In 2009, the New York State Department of Health supplemented this system with its Sentinel Hospital Program (SHP). This includes data from six large hospitals serving parts of New York not included in the EIP that reported the number of patients who were admitted with the chief complaint of ILI and submit up to 20 respiratory specimens per week for confirmatory pH1N1 testing by PCR. ILI was defined as temperature of >37.8 C and cough and/or sore throat [27].

CDC collects timely, influenza-associated mortality data through two different systems. The 122 Cities Mortality Reporting System receives data from 122 cities throughout the U.S. on the total number of death certificates received and the number of those for which pneumonia or influenza was listed as the underlying or contributing cause of death. The Influenza-Associated Pediatric Mortality Surveillance System tracks laboratory-confirmed influenza-related deaths among children under 18 years of age based on reports submitted by state, local, and territorial health departments [2]. In addition, starting in August 2009 CDC requested that states submit aggregate data on hospitalizations and deaths due to influenza using either a laboratory-confirmed or syndromic case definition through its new Aggregate Hospitalizations and Deaths Reporting Activity (AHDRA) reporting system.

Another innovation in 2009 was the development and implementation of a module for CDC's Behavioral Risk Factor Surveillance System (BRFSS) to provide data on ILI in the community. Between September 2009 and March 2010, more than 250,000 adults and children responding to this ongoing state-based population survey were asked about their own ILI, which was defined as the presence of fever with cough or sore throat, in the previous month [23].

The geographic distribution of influenza activity across the United States is reported weekly by state and territorial epidemiologists. States report influenza activity as no activity, sporadic, local, regional, or widespread [2]. In addition, many of the surveillance data systems described above provide regional or state level data, which are available in CDC's Weekly Influenza Surveillance Report FluView.

The final data sources for our analysis come from Google. Google Flu Trends is based on the number of queries using a specific set of flu-related search terms that they have found to be related, in the aggregate, to the number of people who have flu symptoms [33]. Google Insights for Search is a more generic tool for quantifying the relative number of Google searches on a certain topic on a weekly basis [34]. For our analysis we used the number of internet searches for “swine flu” (“H1N1” was similar during the Fall of 2009 but was uncommon in the Spring). We also restricted the analysis to searches for swine flu news items. The information environment, of course, is far more complex than these indices can represent, but they provide an objective general sense of public interest and concern.

Because there are no data that describe the actual rates of pH1N1 infection in the United States, or its consequences, we adopted a “triangulation” approach [35] in which multiple contemporary data sources, each with different expected biases, are compared to identify time patterns that are likely to reflect biases versus those that are more likely to be indicative of actual infection rates. This public health systems research approach is grounded in the understanding that each of these surveillance systems is a production process. From this perspective, surveillance data are the result of a series of decisions made by patients, health care providers, and public health professionals about seeking and providing health care and about reporting cases to health authorities. Outpatient, hospital-based, and ED surveillance systems, for instance, all rely on individuals deciding to present themselves to the health care system based on their interpretations of their symptoms. Even the number of Google searches and self-reports of ILI in the BRFSS survey can be influenced by their interpretation of the seriousness of their symptoms. Virologic surveillance and systems based on laboratory confirmations all depend on physicians deciding to send samples for testing. Moreover, every element of this decision-making is influenced by the informational environment (i.e. media coverage, implementation of active surveillance), processing and reacting to the information on an individual level (i.e. the health care seeker's self-assessment of risk, incentives for seeking medical attention and self-isolation, the health care provider's ordering of laboratory tests), and technical barriers (i.e. communication infrastructure for data exchange, laboratory capacity), all of which change constantly.

### Conclusions

The U.S. public health surveillance response to pH1N1 offers much to admire. Together with surveillance activities in Mexico, and benefiting from advances in laboratory capacity and notification systems introduced in the previous decade, epidemiologists relatively quickly identified and characterized a new pathogen, allowing the CDC and shortly afterwards the WHO to issue alerts about the emergence of a new pandemic strain, triggering a rapid global public health response to H1N1 [8]. These alerts triggered pandemic influenza plans that had been prepared in recent years, which focused initially on surveillance activities, including the development of a case definition and testing procedures, and non-pharmaceutical control measures. A variety of existing influenza surveillance systems were supplemented with newly developed *ad hoc* systems to provide timely information to guide policy makers and monitor the public health response [2].

However, although not definitive, our analysis of the of the public health surveillance systems that provided situational awareness in the United States calls into question two aspects of the conventional wisdom about the 2009 H1N1 pandemic. In particular, children and young adults might not have experienced higher risks of suffering severe consequences as some thought. Furthermore, apparent patterns in the timing and relative size of the Spring and fall wave, and of geographical differences in timing of the fall wave, seem to depend in large part on perceptions of what was happening. Although the extent of the biases suggested by this analysis cannot be known precisely, the analysis identifies underlying problems with surveillance systems – in particular their dependence on patient and provider behavior, which is influenced by a changing information environment – that could limit situational awareness in future public health emergencies.

These problems are particularly germane for syndromic surveillance systems, which are highly dependent on individuals' decisions to stay home from school, self-treat, or seek health care, and thus on changes in the information environment. The earliest appearance of the pandemic did not trigger a quantitative alert in any of these systems, either in Mexico or in the United States [22]. Lipsitch and colleagues report both instances in which syndromic surveillance was thought to be effective for situational awareness and others in which it was misleading [22]. Our analysis of the pH1N1 experience and another comparing syndromic surveillance systems in the Fall of 2011 at two universities [36] do not support the PCAST report's enthusiastic endorsement of this approach for situational awareness [3].

Similarly, this analysis has implications for the on-going redesign of the CDC BioSense program, which was originally mandated in the *Public Health Security and Bioterrorism Preparedness Act of 2002* to establish an integrated national public health surveillance system for early detection and rapid assessment of potential bioterrorism-related illness [37]. Current re-design efforts focus on building a network of syndromic surveillance systems and a community of surveillance practitioners who share data and interpretations, including enhancements to the data acquisition process to improve data quality and timeliness [38]. In this context as in other information technology discussions, data quality and timeliness refer to the transfer of information from the source to the point of analysis, that is efforts to ensure that data from hospitals or other health care providers is transmitted quickly and without loss of fidelity to BioSense analysts. The proposed enhancements do not address the biases identified in this paper, which affect the quality of epidemiologic information – as opposed to the data – that BioSense produces.

Our analysis is not intended as a criticism of the epidemiologists charged with the immense challenge of tracking the impact of a new pathogen. Rather, this analysis shows how fundamentally difficult the surveillance challenge is, and sheds light on the adequacy of existing surveillance systems for such efforts. As with most novel pathogens, the emergence of pH1N1 was characterized by uncertainty that took weeks to months to resolve. Epidemiologists familiar with the emergence of novel pathogens rightly compare the rapidly evolving facts and scientific knowledge to the “fog of war,” [39], and the United Kingdom's Pandemic Influenza Preparedness Programme has shown how it should be factored into public health preparedness planning [40]. Many emergency preparedness professionals, however, still think in terms of single cases triggering a response in hours or days, and this thinking is reflected in such key public health preparedness documents as CDC's 2011 *Public Health Preparedness Capabilities: National Standards for State and Local Planning* [41]. A more appropriate response would be a plan to step up surveillance efforts early in the event to fill in the uncertainties apparent in the original data.

Moreover, to the extent that future public health crises are characterized by similar unknowns, this analysis of the 2009 H1N1 experience identified a root cause that is likely to be problematical in the future: surveillance systems that depend on patients and providers taking action – to seek care, to send samples for testing, and so on. These actions depend, to some extent, on what people hear in the media and official reports, creating an inherent circularity that is difficult to disentangle. This problem is most prominent in surveillance systems based on reported cases, but also affects everything from syndromic surveillance systems that monitor individuals' decisions to seek health care to virologic surveillance systems that depend on physicians submitting specimens to be tested. This is yet another example of how case-based surveillance systems can lead to biased statistical data [42]. Similarly, Lipsitch and colleagues [22], [43] and Garske and colleagues [44] both show how biased case ascertainment especially early in the pH1N1 pandemic led to over-estimating the case fatality rate.

The optimal solution is to develop population-based surveillance systems that are less dependent on individuals' and their physicians' decisions, and thus less sensitive to the circular effect of the media environment. Lipsitch and colleagues, for instance, have suggested identifying well-defined population cohorts at high risk for pH1N1 infection and ensuring that everyone in that group is tested to avoid biases due to physician decisions about who should be tested [43]. Kok and Dywer review a number of possible designs [45], and Lipsitch and colleagues [22] describe their beneficial effects. Another possibility is to make more timely use of on-going national population-based surveys such as CDC'S Behavioral Risk Factor Surveillance System (BRFSS). This was used in 2009 to provide monthly data on the proportion of the population with influenza-like symptoms, but the results were not published until long after the pandemic wave had passed [23]. New York City mounted a more limited but also more timely telephone survey in May and June, 2009 [46]. With proper planning and statistical techniques, the same survey could be used to produce more timely data. Ultimately, a population-based seroprevalence survey, like those deployed in the United Kingdom [47] and Hong Kong [48] would provide the least biased data on who is at risk for infection as well as temporal and geographic patterns. The benefits of such studies, however, are tempered by practicalities of obtaining informed consent, the unavailability and limitations of early serologic tests, as well as the costs, so Lipsitch and colleagues conclude that a large-scale prepandemic investment would be needed to improve current influenza serologic assay technology sufficiently so that valid serologic tests could be developed quickly at the start of the next pandemic [22].

Other problems sprang from common difficulties in clearly presenting scientific information to policymakers and the public. Epidemiologists were aware of the distinction between the risk of pH1N1 infection and its consequences, yet policymakers developed immunization strategies and priorities predicated on the assumption that children were at high risk of its consequences. Presumably this also reflects a general concern for and need to protect children, but surely population perceptions about children being “at risk” contributed to this. One might argue that epidemiologists cannot control how their data are used, but the standard definition of public health surveillance is the “ongoing, systematic collection, analysis, and *interpretation* of health-related data essential to the planning, implementation, and evaluation of public health practice, *closely integrated with the timely dissemination of these data to those responsible for prevention and control*” (emphasis added) [49].

Beyond considerations of needed surveillance system improvements, this analysis also has implications for how to measure preparedness. For instance, CDC's and the Trust for America's Health's most recent state-by-statement assessments of public health preparedness focus on ensuring that state and local public health laboratories have the *capacity* to respond rapidly, identify or rule out particular known biological agents, and increase the workforce and laboratory throughput needed to process large numbers of samples during an emergency [50], [51]. None of these measures would have ensured that public health surveillance systems could have avoided the biases identified in this analysis and accurately tracked who was at risk for pH1N1 and monitored the development of the pandemic over time and in different geographical areas. Nor did syndromic surveillance or other approaches to harnessing the vast amounts of data in electronic medical records avoid problems with bias in 2009 or could be expected to do so in the future. Although these capacities are clearly necessary, they are not sufficient. Rather, assessing the nation's level of preparedness requires measures of the systems *capability* to accurately track an outbreak and characterize its risks, and to communicate this information clearly to policy makers.

In addition to its substantive contributions, this analysis illustrates the quality improvement (QI) approach called for in the U.S. National Health Security Strategy (NHSS) [52]. Emphasizing processes (chains of events that produce specific outcomes) and systems of people and information, the QI approach refers to a range of specific practices including procedures and system changes based on their effects on measurable outcomes, reducing unnecessary variability in outcomes while preserving system differences that are critical to the specific environment, continuous improvement rather than onetime initiatives, and critical event/failure mode analysis. In general, our analysis is an example of an effort “to collect data on performance measures from real incidents … analyze performance data to identify gaps, [and] recommend and apply programs to mitigate those gaps,” an approach called for in the NHSS Implementation Guide [53].

Learning about public health systems' emergency response capabilities is challenging because actual events are unique, and both the epidemiological facts and the context varies from one community to another. In other words, there is no replication, a centerpiece of the scientific method. In this context, to address the lack of a gold standard to describe the actual trends in the rate of pH1N1 infection and its consequences over time, we adopted a “triangulation” approach that compares multiple data sources, each with different likely biases. We focus specifically on two time periods, the month of May when the Spring peak occurred and the fall wave, which started at the end of August and continued through November. In the Fall, we also focus on the South and the Northeast regions of the United States, which respectively had the earliest and latest fall peaks. Statistical calculations were limited to simple data transformations to ease comparability and graphical analysis. This type of analysis is necessarily qualitative and contextual; rather than serving as a recipe for doing this in other settings, this analysis should be seen as an example that illustrates the concept. Similar analyses of other events will require a different approach.
